# Computational Investigation of Structure and Bonding of In_2_O_3_ Surfaces: Relevance to CO_2_ Hydrogenation

**DOI:** 10.1021/acs.jpcc.5c01539

**Published:** 2025-07-16

**Authors:** Samadhan Kapse, Francesc Viñes, Francesc Illas

**Affiliations:** Departament de Ciència de Materials i Química Física & Institut de Química Teòrica i Computacional (IQTCUB), 16724 Universitat de Barcelona, c/Martí i Franquès 1-11, 08028 Barcelona, Spain

## Abstract

This study utilizes periodic density functional theory (DFT) to investigate the catalytic potential of indium oxide (In_2_O_3_) surfaces, including (111), (110), and (100), for CO_2_ hydrogenation. The analysis evaluates surface stability, chemical bonding, and reducibility, supported by the electron localization function and Bader charge analyses, which reveal the electronic properties of various sites and the influence of oxygen vacancies. This study focuses on the In_2_O_3_ (111) surface, identifying specific active sites that are highly favorable for CO_2_ and H_2_ adsorption. The impact of cobalt (Co) doping and Co_2_O_3_ cluster embedding is analyzed, revealing enhanced charge redistribution and adsorption capabilities, which significantly improve the catalytic performance of the active sites. These findings underscore the stability and catalytic efficiency of Co-modified In_2_O_3_ surfaces for the application of CO_2_ hydrogenation.

## Graphical abstract

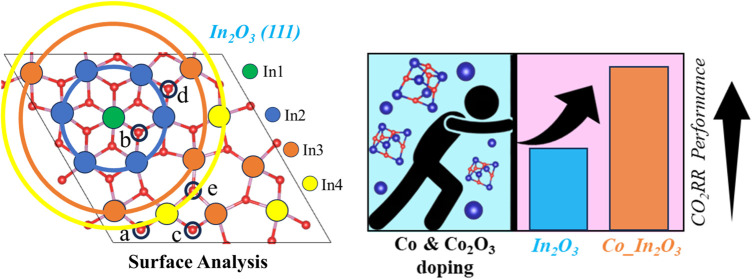

## 1 Introduction

The increase in atmospheric CO_2_ due to anthropogenic activities, mainly from fossil fuel consumption, and driven by economic development and population growth, is a major cause of global warming with severe environmental impacts such as sea level rising, ocean acidification, and extreme weather events. To mitigate these issues, current research accelerated toward CO_2_ capture, storage, and its conversion into valuable chemicals, such as methanol, via hydrogenation using suitable heterogeneous catalysts, especially when using green hydrogen, thus contributing to achieve a carbon-neutral economy.^1,2^ Among the different possibilities, the catalytic conversion of CO_2_ to methanol (CH_3_OH), using renewable hydrogen, offers an interesting and sustainable option as an alternative fuel and energy carrier. This power-to-gas technology, using CO_2_ as a feedstock, has been presented as a promising method for utilizing surplus renewable energy as well.^3^ However, the high stability of CO_2_, poor selectivity, and stability of existing catalysts, together with not completely clear reaction mechanisms, still hinder progress in the field. In the chemical industry, Cu-based catalysts have long been employed for CO_2_ hydrogenation, leveraging their established use in methanol production from syngas,^4,5^ a mixture of CO and H_2_. Despite significant efforts to enhance these catalysts, they continue to face limitations such as deactivation of the copper component.^6^ This challenge highlights the need for advanced catalysts with long-term stability, enhanced activity, and selectivity to achieve efficient CO_2_ hydrogenation.

Previous studies have shown that while catalysts based on oxides and their interfaces with metals do exhibit high methanol selectivity in CO_2_ hydrogenation, further enhancements in catalytic activity are still necessary for practical industrial applications.^7–10^ Among oxide-based catalysts, indium oxide (In_2_O_3_) has demonstrated remarkable potential due to its ability to efficiently adsorb and activate CO_2_ for hydrogenation to CH_3_OH or CO, thereby outperforming conventional catalysts.^11–14^ Theoretical investigations in the framework of density functional theory (DFT) identified oxygen vacancies as the key active sites responsible for CO_2_ adsorption, activation, and the stabilization of critical intermediates.^15–17^ This has provided valuable insights into the structure–activity relationships of these catalysts. In addition to superior activity, In_2_O_3_ offers excellent stability and durability, especially when supported on zirconia, which further boosts methanol production rates.^13^ These combined features of high selectivity, catalytic efficiency, and robustness exhibited by In_2_O_3_ make it one of the most promising catalysts for CO_2_ hydrogenation. However, In_2_O_3_ has shown limited efficiency in activating hydrogen, as its weak H_2_ splitting capability significantly restricts the rate of CO_2_ conversion.^18^ To overcome this problem, various metal–In_2_O_3_ interactions were explored, revealing enhancements in the hydrogen dissociation capabilities of In_2_O_3_-based catalysts.^19–23^ It has also been reported that In_2_O_3_(110) and In_2_O_3_(111) surfaces promote methanol synthesis via the formate pathway,^24,25^ although the reverse water gas shift (RWGS) and the CO hydrogenation reaction are favored in In_2_O_3_-supported metal catalysts.^26,27^ Also, Frei et al.^28^ reported that the formation of active interfacial sites with finely dispersed InNi_3_ alloy phases on the oxide surface promotes effective homolytic splitting of hydrogen.

From the computational side, DFT studies by Yu et al.^29^ and Li et al.^30^ predicted that the oxygen-defective In_2_O_3_(110) surface offers superior catalytic performance. Subsequent investigations have explored the properties of In_2_O_3_(110) for the CO_2_ hydrogenation. However, this is not the lowest energy surface and, consequently, it is not the most exposed one. In fact, In_2_O_3_(111) is the thermodynamically most stable surface of this material^31,32^ and has been the subject of studies concerning CO_2_ hydrogenation.^33–35^ A recent study has demonstrated that Co-supported In_2_O_3_ significantly improves both the activity and the selectivity for methanol production,^36,37^ presenting a promising alternative to costly Pd- and Pt-supported In_2_O_3_. Even more recently, doping In_2_O_3_ with different amounts of Co has also been shown to boost CO_2_ photothermal conversion.^38^ However, detailed insights into the geometric and electronic structures as well as the reactivity of pristine, defective, and doped In_2_O_3_ surfaces remain insufficiently explored.

In this work, we present a systematic DFT-based study of the properties of the stoichiometric In_2_O_3_ (100), (110), and (111) surfaces with a focus on their potential for CO_2_ hydrogenation. Key properties, such as surface energies, work function, electron localization function, and Bader charge analysis, were examined. For the most stable (111) surface, a more detailed study was conducted, focusing on symmetry aspects, the potential active sites, the stability of surface oxygen vacancies, Co atom doping, and the embedding of Co_2_O_3_ clusters as in a solid solution. Finally, we studied the potential sites and configurations of this surface for H_2_ and CO_2_ adsorption, which is a key step in the formation of CO_2_ hydrogenation. The influence of Co modification on the In_2_O_3_ surface in altering the active sites for the RWGS reaction is also evaluated. This research aims to enhance understanding and systematically develop highly efficient Co-doped In_2_O_3_ structures for catalyzing CO_2_ hydrogenation.

## 2 Surface Models and Computational Details

The In_2_O_3_ surfaces have been represented by appropriate periodic models, as described in detail below. The models have been built using the stable cubic In_2_O_3_ polymorph from the Inorganic Crystal Structure Database (ICSD), which is available through the Materials Project platform. This cubic bixbyite-type In_2_O_3_ phase belongs to the *I*a3̅ space group and the unit cell consists of 80 atoms in total, 32 metal atoms and 48 oxygen atoms (cf. Figure 1). The bulk crystal structure was first optimized from first-principles DFT-based calculations carried out with the Vienna *Ab initio* Simulation Package (VASP)^39^ using the Perdew–Burke–Ernzerhof (PBE) functional within the generalized gradient approximation (GGA).^40^ The Kohn–Sham equations were solved iteratively to achieve self-consistency, using a plane-wave basis set with a cutoff energy of 415 electronvolts (eV) to expand the valence electron density, whereas the projector augmented wave (PAW)^41^ method was used to account for the interaction between the valence electron density and the atomic cores. Convergence criteria were set at 10^–5^ eV for the total energies and 0.01 eV/Å for the forces.

**Figure 1:**
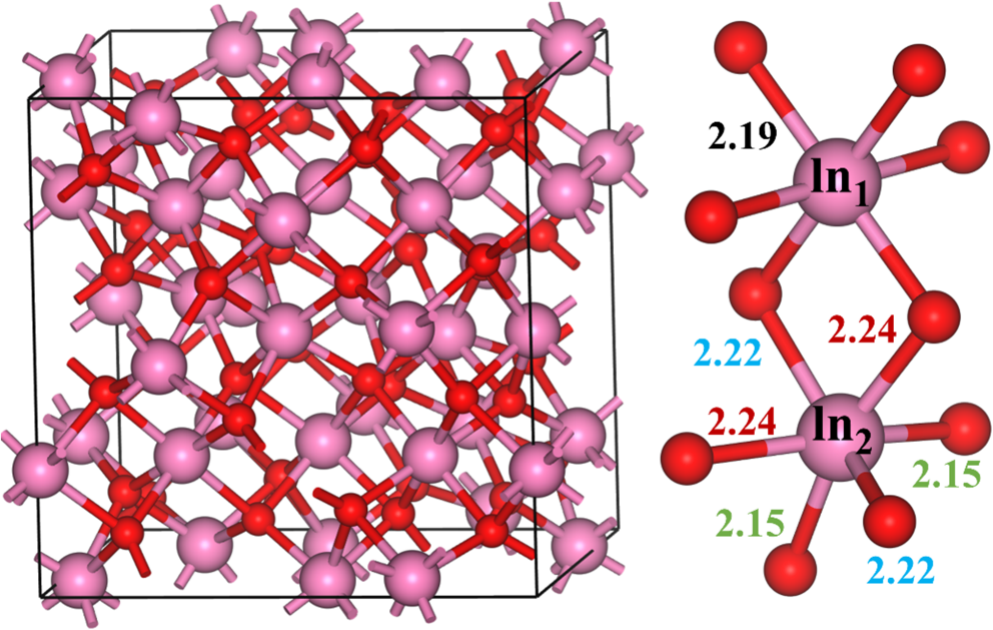
Unit cell of the cubic In_2_O_3_ crystal structure (left) image, and vicinal In–O bond distances, given in Å, for the two types of In atoms, In_1_ and In_2_. The pink and red color spheres indicate indium and oxygen atoms, respectively.

From the bulk crystal structure, slab models of different thicknesses were constructed for the (111), (110), and (100) directions to obtain the different surface models. The In_2_O_3_ surfaces were modeled as two-dimensional slabs with periodic boundary conditions, incorporating a 20 Å vacuum along the vacuum (*z*) direction to prevent interactions between repeated images. A convergence test using different grids of special **k**-points^42^ ensured a total energy convergence within 1 meV, when the Brillouin zone for the slab models was sampled using a 3 × 3 × 1 Monkhorst–Pack grid. Additionally, denser Monkhorst–Pack grids of 9 × 9 × 1 were employed to accurately characterize the electronic structure of the optimized models.

The surface energy, γ, is a fundamental property determining the thermodynamic stability of the different facets. Hence, knowing γ is essential to study surface phenomena such as surface roughening, segregation, catalytic activity, and the equilibrium shape of crystals.^43^ The surface energy is defined as the energy per unit area needed to create a surface relative to bulk material and is calculated as follows

1 $$\gamma =\frac{E_{\mathrm{slab}}-E_{\mathrm{bulk}}}{2\cdot A}$$

where *E*_slab_ is the slab PBE total energy, *E*_bulk_ is the PBE energy of an equivalent number of bulk In_2_O_3_ units, and *A* refers to the surface area formed on each side of the slab.

Another property of interest is the oxygen vacancy formation energy, *E*_O_vac__, here estimated as usually^17,44^ as in eq 2,

2 $$E_{O_{vac}}=\{\left(E_{{In}_{2}O_{3-1}}+\frac{1}{2}E_{O_{2}}\right)-E_{{In}_{2}O_{3}}\}$$

where *E*_In_2_O_3__ is the PBE energy of corresponding In_2_O_3_ surface model, *E*_In_2_O_3 – 1__ is the PBE energy of the same surface model but with one O vacancy, and *E*_O_2__ stands for the PBE energy of an O_2_ molecule in the gas phase, in its triplet ground state, calculated within a large box of 15 × 15 × 15 Å^3^ dimensions, and is estimated here as −10.77 eV.

## 3 Results and Discussion

### 3.1 Properties of the Stoichiometric In_2_O_3_ Surface

We first comment on the PBE-optimized bulk structure of In_2_O_3_, which has an optimized lattice parameter of 10.227 Å, in agreement with reported works that used the same method.^24,45^ In the most stable polymorph of In_2_O_3_, namely, the body-centered cubic (bcc) bixbyite crystal structure,^46^ two types of indium atoms are present, denoted as In_1_ and In_2_ in Figure 1. The In_1_ site is attached to six equivalent oxygen atoms with the same bond length (2.19 Å) and In_2_ is connected to six oxygen atoms with three different values of bond lengths (2.15, 2.22, and 2.24 Å). From the three considered low Miller index surfaces, note that the (100) surface is polar and can exhibit either oxygen or indium terminations; these two will be denoted as (100)-O and (100)-In, respectively. On the other hand, the (110) and (111) facets are nonpolar in nature.^31^ Note also that the stacking of atomic layers along each direction perpendicular to the surface is not easy to define, as oxygen atomic layers can be very close. To avoid misunderstandings, we define the slab thickness in terms of the number of In layers. The different surface models are represented in Figure S1 of the Supporting Information (SI).

To obtain the best models to represent the different surfaces, we studied slab models with varying thicknesses, including up to seven In atomic layers for the (111) surface and eight In atomic layers for the (110) and (100) surfaces. All generated using optimized bulk In_2_O_3_ structure as shown in Figure S2 of SI. In all of these models, we allowed the two topmost In atomic layers and the O atoms directly coordinated to them to relax, while keeping the remaining In and O layers fixed to provide a bulk environment to the atoms in the underlying layers (cf. Figure S3 of SI). To assess the convergence of the electronic structure with slab thickness, we focus on the Bader charge of one In and one O atom on the surface of the considered (100), (110), and (111) models. Figure S2 of SI shows that for slabs with more than five In atomic layers, these charges do not vary, thus revealing that a slab model with fewer atomic layers can lead to biased results. Therefore, all results reported in the present work are obtained with slab models with five In layers, schematically shown in Figure 2.

**Figure 2:**
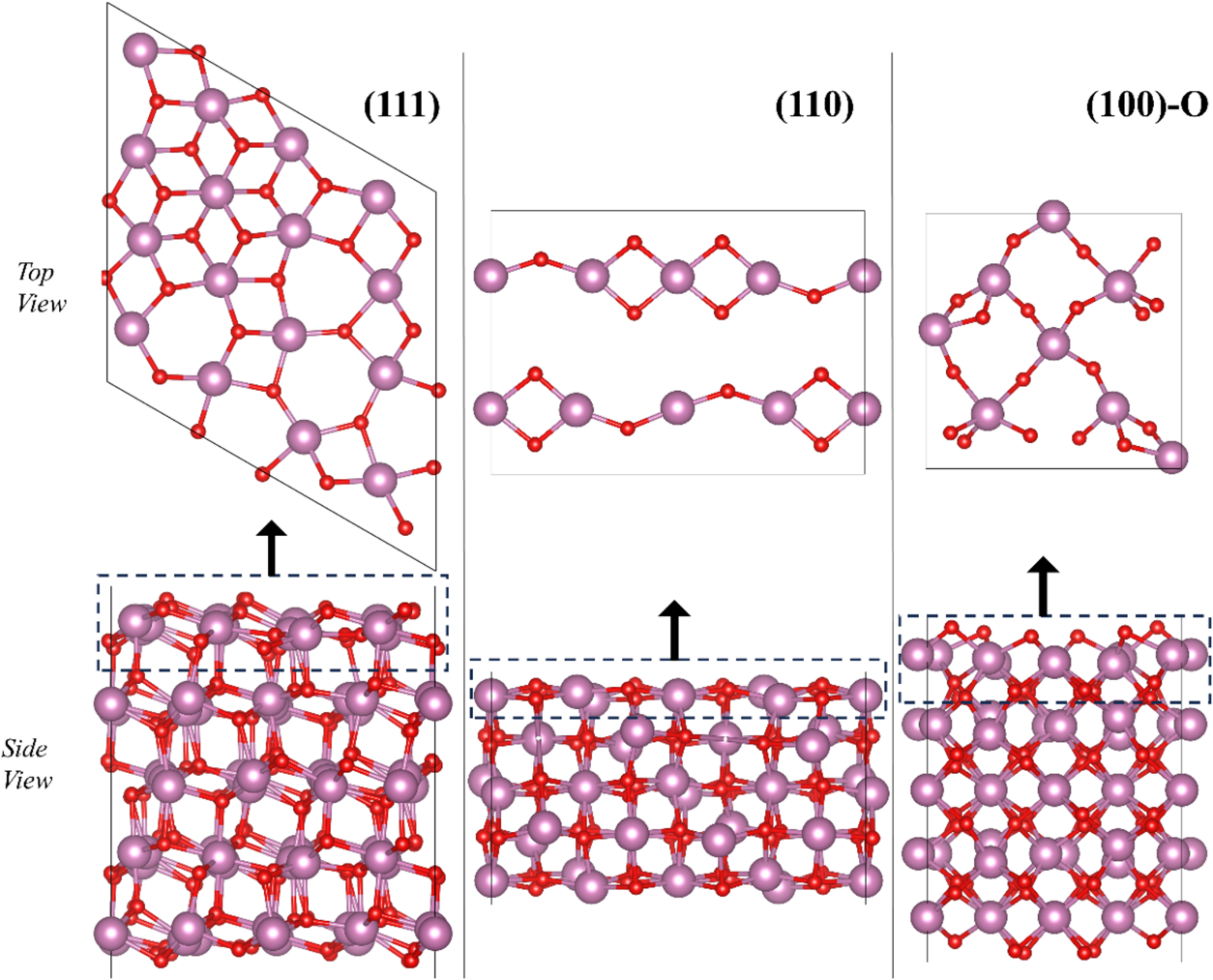
Three slab models used to represent the (111), (110), and (100)-O In_2_O_3_ surfaces. Color code as in Figure 1.

To unveil the relative stability of the In_2_O_3_ surfaces, we computed the surface energy γ of the (111), (110), (100)-O and (100)-In models. Since this quantity is sensitive to the surface relaxation, γ was computed for three different situations. These are a slab with only the top two In layers and their corresponding O layers are relaxed (Top-Relaxed or TR) as the one discussed above to inspect the effect of the thickness plus a model with the two top and two bottom In layers and their corresponding O atomic layers are relaxed (Top Bottom Relaxed or TBR), and finally, a fully relaxed (FR) slab. For the (111) surface, these situations are shown in Figure S4 of SI, whereas the calculated γ values for each case in the (111), (110), (100)-O and (100)-In surfaces are summarized in Table 1. Regardless of the surface model used, the (111) surface is always the most stable, followed by (110), (100)-O, and (100)-In being less stable. On the other hand, both O- and In-terminated (100) surfaces show significantly higher surface energies, with the (100)-In surface being the least stable. It is also noteworthy that the γ for the slab, where both top two and bottom two ionic layers were optimized (TBR), closely matches with values reported in previous studies.^31^ As expected, the lowest γ values correspond to the FR model and the highest to the TR model.

**Table 1:** Surface Energy, γ, and Work Function, ϕ, of FR, TBR, and TR Models of the (111), (110), (100)-O, and (100)-In Surfaces

	γ (J/m^2^)			ϕ (eV)		
surfaces	FR	TBR	TR	FR	TBR	TR
(111)	0.71	0.85	0.86	5.80	5.84	5.64
(110)	1.02	1.05	1.16	5.42	5.38	5.30
(100)-O	1.78	1.81	2.10	5.90	6.78	6.89
(100)-In	1.55	2.05	2.25	5.57	5.13	5.18

To further understand the electronic properties of these surfaces, we also computed the work function, ϕ, and electron localization function (ELF). Mostly, the ϕ follows the trend: (100)-O > (111) > (110) > (100)-In. The ϕ values for all models are listed in Table 1, and the observed trend in ϕ across surfaces is consistent with previously reported results, e.g., the O-terminated (100) surface, with negatively charged O atoms, has a dipole opposed to the electron extraction, increasing ϕ, while the opposite happens on the (100)-In surface.^31^ This analysis suggests that modifying surface morphology provides a potential approach for tuning the ϕ, which could be beneficial for various applications such as optoelectronics or in photocatalysis.

As far as the ELF analysis is concerned, we note that it allows visualization of the electron pair distribution and chemical bonding among surface atoms. Among the latter, the (100)-O is more stable and will be the one considered in the rest of this work. The ELF contour maps for the (111), (110), and (100)-O surface models are presented in Figure 3. The ELF values, ranging from 0 to 1, represent electron pair distributions from complete delocalization to full localization. Across all surfaces, electron pair localization is consistently observed around the oxygen atoms, as expected from the quite high ionic character of the chemical bond in these oxides.^47^ However, the ELF values vary depending on the specific slice of the surface being analyzed. Notably, the (110) surface with all atoms on the same plane exhibits higher ELF values and stronger electron localization around the oxygen atoms in comparison to the other surfaces. Also, we present ELF of the (111) surface with a two-dimensional (2D) contour slice focusing on the oxygen atoms at the *b* site, located at the top of the (111) surface (cf. Figure S5 of SI). The results show that the electron pair density is highly localized around these oxygen atoms. This electron pair localization on oxygen at the *b* site may play an important role in influencing adsorption as well as activation of molecules near such a site on the (111) surface.

**Figure 3:**
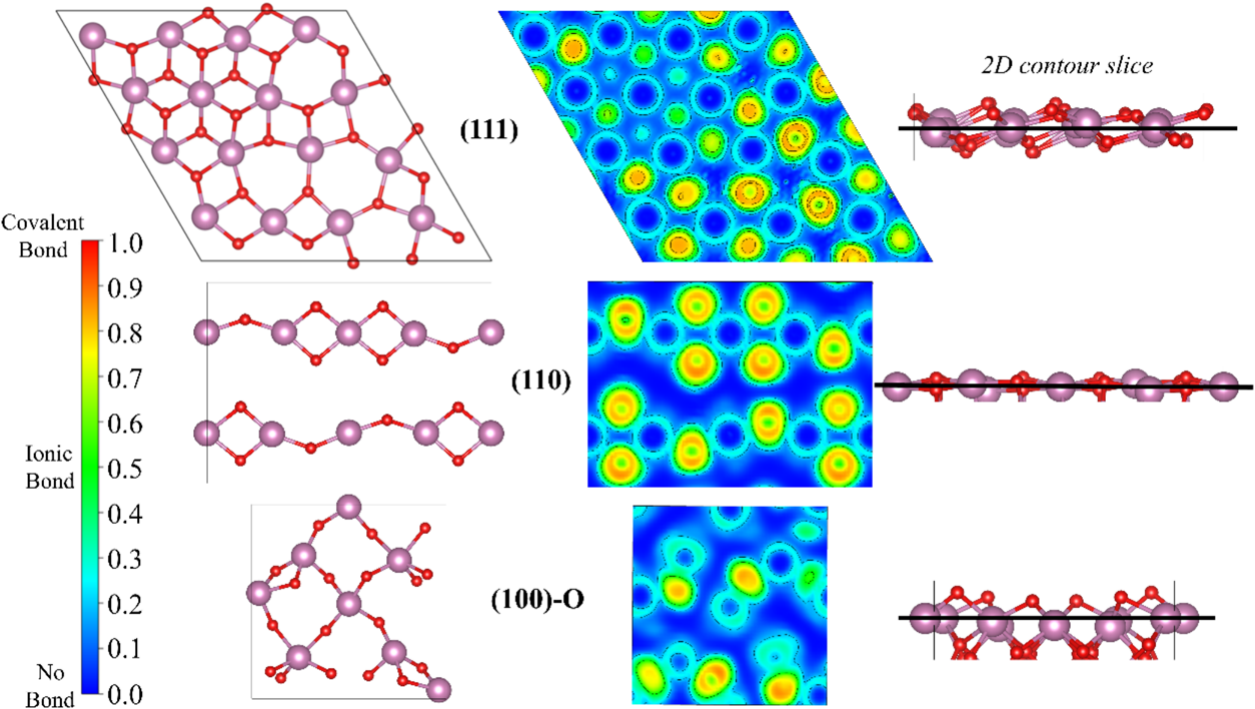
Top, middle, and bottom panels display the atomic structure and ELF cuts of the (111), (110), and (100)-O surfaces. Atomic color coding as in Figure 1.

Numerous previous studies, both experimental and theoretical, have emphasized the potential significance of oxygen vacancies in governing catalytic reactions on In_2_O_3_.^48–51^ However, recent investigations have revealed that the O 1*s* core-level shift (CLS) peaks should be attributed to hydroxyl groups instead of oxygen vacancies and that surface oxygen vacancies are not present on stoichiometric In_2_O_3_(111) in ultrahigh vacuum environment.^52,53^ To further systematically investigate this issue, we computed the formation energy of oxygen vacancies (*E*_O_vac__) at various surface sites of the (111), (110), and (100)-O surfaces, as shown in Figure 4. From the calculated results, also collected in Figure 4, it appears that the (100)-O surface is the most reducible, with quite low *E*_O_vac__ values in the 0.46–0.56 eV range, indicating rather easy reducibility. In contrast, the O atoms of the (111) surface exhibit quite high *E*_O_vac__ values ranging from 1.62 to 2.59 eV for the *b* and *a* sites in Figure 4, respectively, making this surface hardly reducible. In the case of the (110) surface, the lowest *E*_O_vac__ is 1.30 eV corresponding to the *b* site, while the highest (2.08 eV) occurs at the *d* site, the values trend being consistent with previously reported findings.^15,54,55^

**Figure 4:**
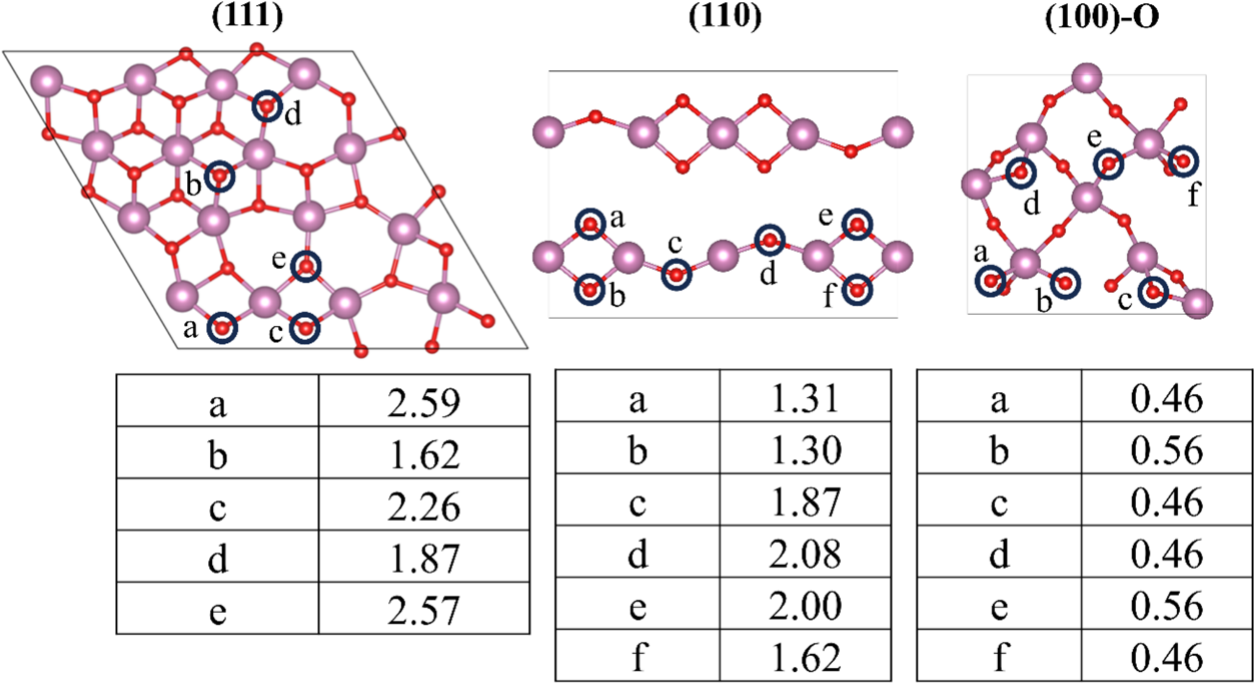
Oxygen vacancy formation (*E*_O_vac__, in eV) for all of the possible inequivalent oxygen sites on the (111), (110), and (100)-O In_2_O_3_ surfaces. Atomic color coding as in Figure 1.

### 3.2 Adsorption of CO_2_ and H_2_ on the Most Stable Stoichiometric and Co-Modified Surface

The rest of this study focuses on the In_2_O_3_(111) surface, as due to its low surface energy, it will be more exposed in catalysts based on this material. First, we analyze the possible active sites on the stoichiometric In_2_O_3_(111) surface for CO_2_ and H_2_ adsorptions, which is a potential step in the CO_2_ hydrogenation process. Figure 5 illustrates the symmetry of indium atoms on this surface. Our analysis revealed four distinct indium sites, labeled In1, In2, In3, and In4, and correspondingly five different oxygen sites also exist, labeled *a*, *b*, *c*, *d*, and *e*. Note, however, that several In–O and O–O configurations are possible on the sites of this surface for the adsorption of CO_2_ and H_2_ molecules. Therefore, all possible combinations of In and O sites were sampled for their potential to adsorb these molecules. The computed adsorption energies for the adsorption of CO_2_ and H_2_ on these active sites are summarized in Figure 6. Note also that certain configurations that are unable to adsorb CO_2_ and H_2_ were excluded from consideration. The lowest adsorption energy for H_2_ was found to be −2.38 eV, while for CO_2_ it was −0.38 eV only. These are highlighted with a blue color in Figure 6. The *b* oxygen sites are favorable for H_2_ adsorption, and both In2 and *b* sites are preferred for CO_2_ adsorption. For adsorbed CO_2_, the carbon atom binds to the *b* site, while the oxygen atom attaches to the In2 site. This study reveals that the oxygen and indium atoms near the In1 site are highly active, a finding also supported by ELF as shown in Figure S5 in SI. The potential adsorption sites for CO_2_ and H_2_ on this surface provide a valuable basis for further investigation into CO_2_ hydrogenation.

**Figure 5:**
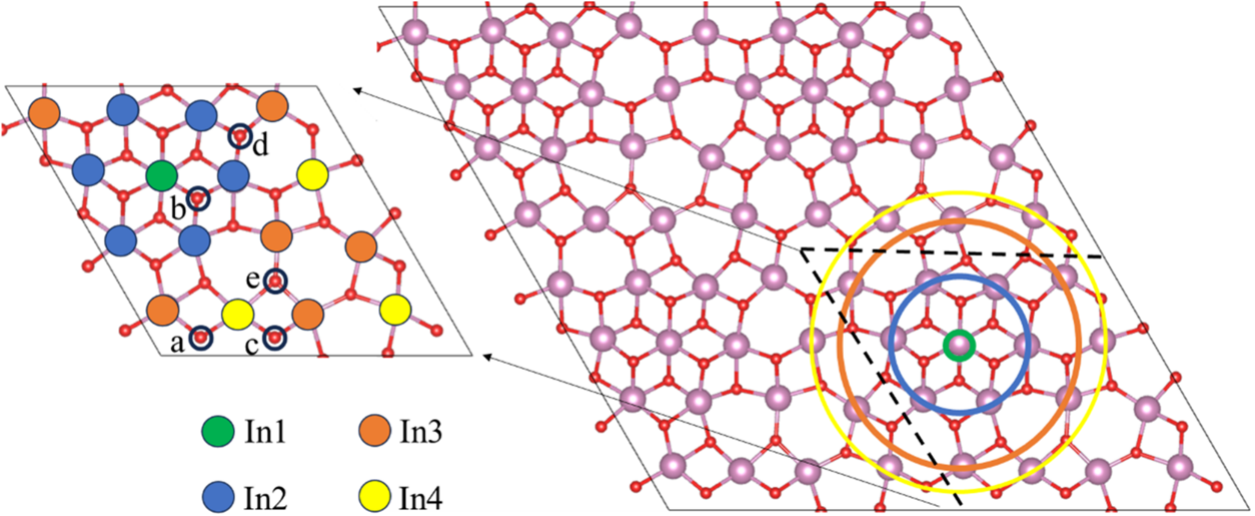
Symmetry of In atoms and oxygen atoms on the (111) surface of In_2_O_3_. Atomic color coding as in Figure 1. The circles denote the coordination spheres.

**Figure 6:**
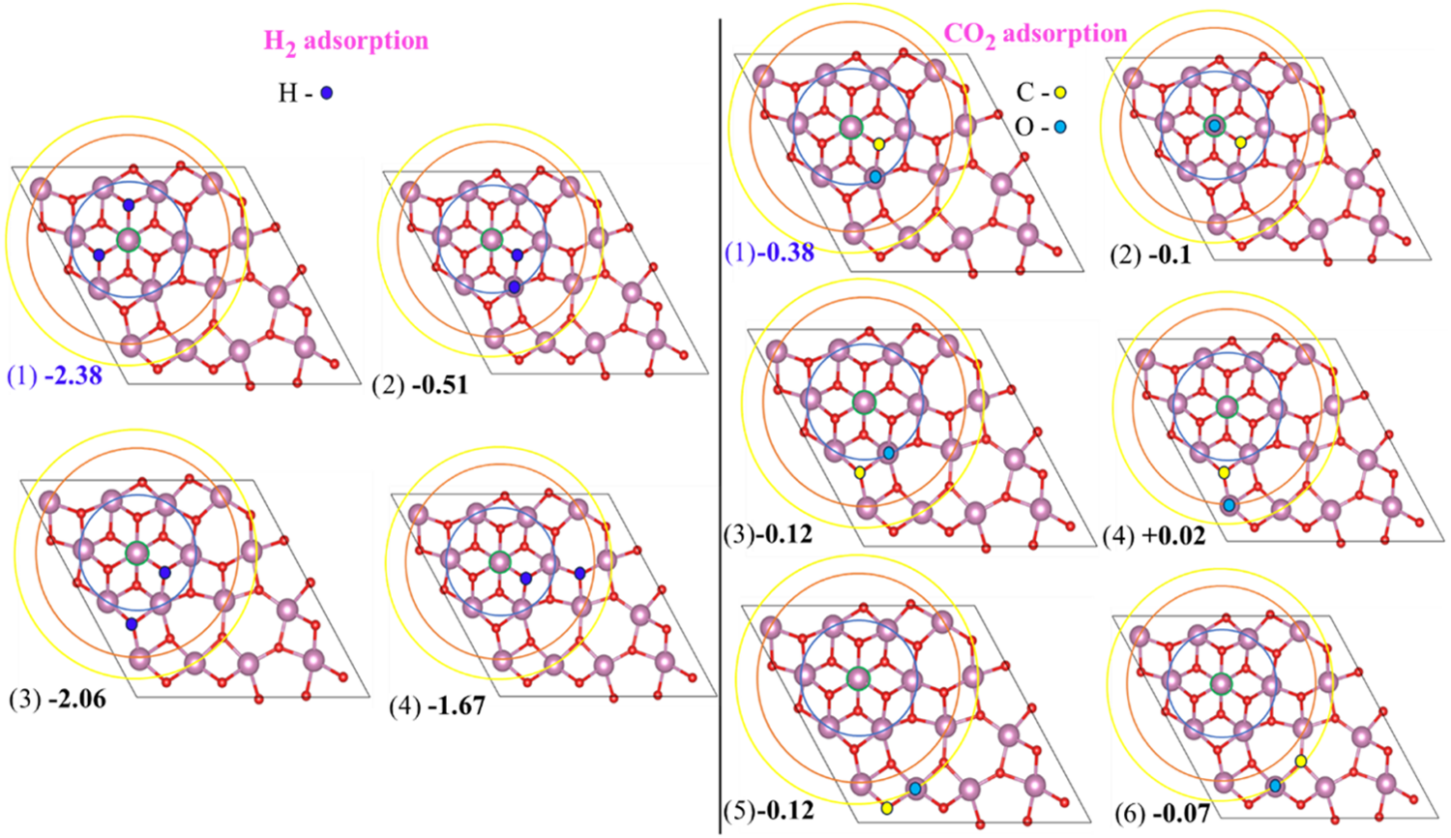
Possible In–O and O–O configurations for H_2_ adsorption (left panels) and CO_2_ adsorption (right panels). All values are given in eV. Circles indicate the coordination shells. Atomic coloring as in Figure 1.

The low CO_2_ adsorption energy on the most exposed In_2_O_3_(111) surface triggered studies in which the material is doped. In particular, recent studies have shown that incorporating Co into In_2_O_3_ enhances its activity and selectivity for methanol production.^36,54,56^ Building on our previous analysis of the structural, energetic, and electronic effects of Co doping in bulk In_2_O_3_,^48^ we extend our investigation to the Co-doped In_2_O_3_(111) surface. To this end, we first calculated the energy required to replace an In atom by a Co atom at four potential surface sites and one In atom at the middle of the slab (cf. Figure S6 of SI). Our results indicate that the In_1_ site is the preferred location for Co doping, as it exhibits the lowest energy cost. Second, doping the Co atom below the surface is more challenging due to its higher formation energy compared with surface sites. However, it is worth noting that Co doping slightly increases *E*_O_vac__ making this surface less reducible.

Apart from isolated Co atoms, we also considered the situation where a Co_2_O_3_ cluster is embedded in the In_2_O_3_(111) surface (Co_2_O_3_/In_2_O_3_), considering various possible configurations. This is triggered by recent experimental and theoretical findings suggesting that, upon increasing the Co concentration, this type of cluster is likely to be formed.^48^ To identify the most stable structure, we systematically modeled all plausible configurations of a Co_2_O_3_ cluster embedded in the In_2_O_3_ (111) surface, as illustrated in Figure S7 of SI. A total of seven distinct Co_2_O_3_/In_2_O_3_ configurations are considered and labeled A–G, based on the substitution of three inequivalent surface indium atoms and three subsurface In atoms. These three surface atoms in each configuration are defined with triangles of different colors. We computed the formation energy for these configurations to compare their stability and to select the best Co_2_O_3_/In_2_O_3_ model. We found that the A configuration is the most stable configuration with comparatively the lowest formation energy (cf. Figure S8 of SI). Therefore, we considered this model for further investigation (Figure 7).

Interestingly, CO_2_ and H_2_ adsorption energies increase in the following order: In_2_O_3_ < Co doping < Co_2_O_3_ cluster. The corresponding values of adsorption energies of CO_2_ are −0.38, −0.41, and −0.56 eV, and for H_2_ are −2.38, −2.59, and −2.71 eV, respectively. To unveil the Co and Co_2_O_3_ effects on active sites, we analyzed the electronic properties such as ELF, Bader charge, and work function for these models. From the ELF analysis, it is seen that, upon Co and Co_2_O_3_ cluster doping, the electron pair localization at the *b* oxygen site decreases (cf. Figure S9 of SI), indicating a disruption in the ionic bonding contributions. Since, as commented above, sites In2 and *b* are important for H_2_ and CO_2_ adsorption, we computed their Bader charges to better understand the effect of Co doping and the Co_2_O_3_ cluster on this surface. We found that the Bader charge of the In2 site is almost unchanged as it decreases from 1.80 *e* in In_2_O_3_ to 1.79 *e* after Co doping, and to 1.78 *e* in the Co_2_O_3_ cluster containing model. In contrast, the charge on the oxygen at the *b* site becomes less negative going from −1.14 *e* in the stoichiometric (111) surface to −0.98 *e* when doped with isolated Co atoms, to −0.68 *e* for the surface with the embedded Co_2_O_3_ cluster. The changes are small for In sites but noticeable for O sites, and in any case, the trend is clear and also consistent with the ELF interpretation discussed above. Therefore, one can safely conclude that the charge redistribution around In2 and *b* active sites after Co doping and Co_2_O_3_ cluster embedded in the (111) surface increases the adsorption energy of CO_2_ and H_2_. Finally, we comment on the work function values that also show a decreasing trend from 5.64 eV for pristine In_2_O_3_(111) to 5.42 eV for Co doping and 5.33 eV for the embedded Co_2_O_3_ cluster. This result indicates that electron transfer to CO_2_ is easier in the doped systems, resulting in improved adsorption energy.

### 3.3 CO_2_ Hydrogenation Mechanism on Stoichiometric and Co-Modified In_2_O_3_ Surface

In this section, we examine the most favorable active sites of the In_2_O_3_ surface for CO_2_ hydrogenation, along with the most stable models of Co-doped and Co_2_O_3_-embedded In_2_O_3_ surfaces. Within the context of CO_2_ hydrogenation, our focus is on investigating the reverse water gas shift (RWGS) reaction, namely, CO_2_^(g)^ + H_2_^(g)^ → CO^(g)^ + H_2_O^(g)^. To this end, we analyzed the potential energy surface for plausible reaction steps on the different models of the considered In_2_O_3_ surfaces. The reaction starts from the clean surface and reactants in the gas phase, with energy (*E*_1_) defined as

3 $$E_{1}=E_{\mathrm{Surf}}+E_{{\mathrm{CO}}_{2}}^{\left(g\right)}+E_{H_{2}}^{\left(g\right)}$$

Next, we consider CO_2_ adsorption on the surface at the *b* oxygen site as shown in Figure 6. The energy of this configuration is

4 $$E_{2}=E_{{\mathrm{CO}}_{2}*}+E_{H_{2}}^{\left(g\right)}$$

where *E*_2_ is the total energy of CO_2_ adsorbed at surface and the H_2_ molecules in the gas phase. Finally, we assume that H_2_ dissociates directly at the surface with the H atoms adsorbed on two oxygens at the *b* site as shown in Figure 6. This is justified by the fact that oxide surfaces are easily hydroxylated as well as from a computational study indicating that H_2_ dissociation at In_2_O_3_(111) and In_2_O_3_(110) surfaces involves relatively small energy barriers of ∼0.3 eV only.^55^ The total energy of the resulting situation can be written as

5 $$E_{3}=E_{{\mathrm{CO}}_{2}*}+E_{H*}+E_{H*}$$

where *E*_3_ is now the total energy of the surface with a CO_2_ molecule and two H atoms adsorbed on the surface. In the proposed mechanism, the next step involves one of the H* atoms reacting with the neighboring CO_2_* leading to adsorbed CO* and OH* situation. The energy (*E*_4_) of this situation is

6 $$E_{4}=E_{\mathrm{CO}*}+E_{\mathrm{OH}*}+E_{H*}$$

In the next step, CO* desorbs from the surface and becomes a CO^(g)^ molecule so that the corresponding energy is

7 $$E_{5}=E_{\mathrm{CO}}^{\left(g\right)}+E_{\mathrm{OH}*}+E_{H*}$$

where *E*_5_ is the total energy of the CO molecule in the gas phase plus that of the surface model containing OH* and H* situation. In the final, OH* and H* will react, leading to H_2_O that desorbs in the gas phase. Therefore, the energy (*E*_6_) of this situation is

8 $$E_{6}=E_{\mathrm{Surf}}+E_{\mathrm{CO}}^{\left(g\right)}+E_{H_{2}O}^{\left(g\right)}$$

The total energy profile of the RWGS reaction for the In_2_O_3_, Co-doped In_2_O_3_, and Co_2_O_3_/In_2_O_3_ models is illustrated in Figure 8. The results clearly indicate that the presence of Co on the In_2_O_3_(111) surface stabilizes the adsorbed species, either reactants or products. Moreover, the stabilization is larger for the case of the Co_2_O_3_ cluster embedded in In_2_O_3_ which has important implications. In fact, from the well-known Brønsted–Evans–Polanyi (BEP) relationships,^57,58^ commonly used as a descriptor in computational heterogeneous catalysis,^59–62^ one would expect that the overall stabilization of the energy profile results in a lowering of the transition state energy of the involved steps and thus offers an explanation to the observed catalytic behavior of Co-modified In_2_O_3_ toward CO_2_ conversion.^38^ This conclusion is further supported by the evidence that these BEP relationships, initially reported for reactions on metallic surfaces, also hold for reactions at oxide and carbide surfaces,^63,64^ and that these relationships are not affected by the choice of the exchange-correlation functional.^65^ Note also that, even though the functional used has a known error in describing the thermochemistry of RWGS in the gas phase, this error can be easily corrected.^66^ Therefore, since it affects all three energy profiles equally, the predicted effect of Co on the overall reaction mechanism can be considered as free of this error.

**Figure 7:**
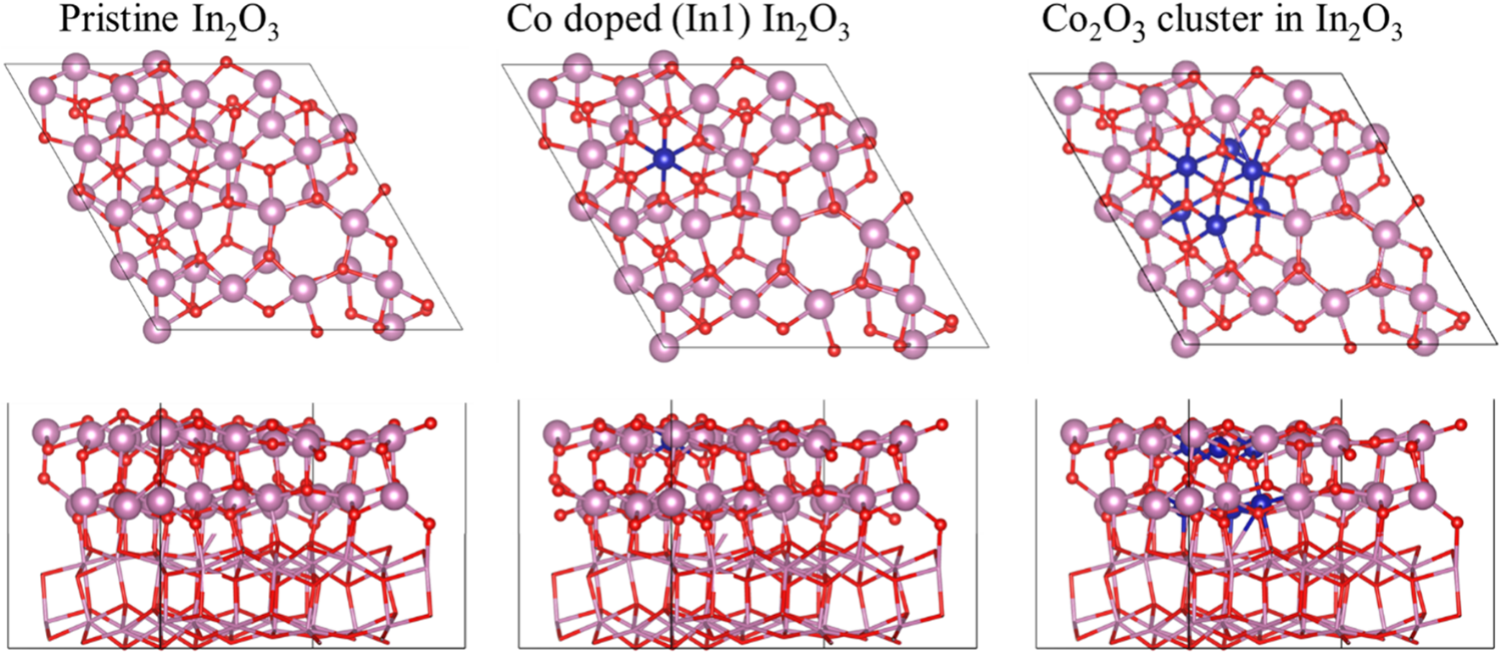
Models used to represent the stoichiometric, Co-doped, and Co_2_O_3_ cluster embedded In_2_O_3_(111) surface. The blue color atom represents the cobalt atom. The rest of the atomic colors as in Figure 1.

**Figure 8:**
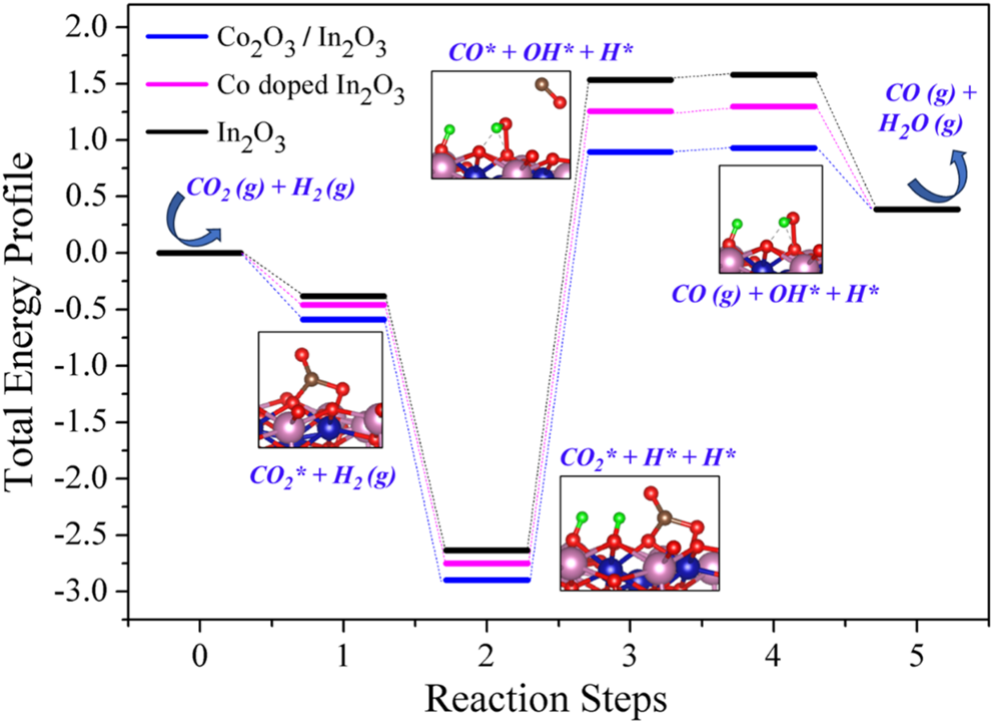
Total energy profile of CO_2_ hydrogenation on (111) surface models of In_2_O_3_, Co-doped In_2_O_3_, and Co_2_O_3_/In_2_O_3_. The labels on the horizontal axis correspond to the energies in eqs 3–8.

## 4 Conclusions

This work presents a systematic periodic DFT study on the properties of low Miller index surfaces of stoichiometric In_2_O_3_ surfaces with special focus on In_2_O_3_(111), as it is predicted to be the most stable one, in agreement with the previous work.^31^ This surface exhibits rather high *E*_O_vac__ values, further confirming its low reducibility, and the electron localization function analysis reveals significant electron pair localization around oxygen atoms, as expected from its strong ionic bonding. Different possibilities of Co doping were also considered, and their potential for CO_2_ hydrogenation was assessed.

For In_2_O_3_(111), we identified and studied four distinct indium sites and six oxygen sites, finding that CO_2_ and H_2_ adsorption is particularly favorable at the In_2_ and *b* sites. Also, Co-doping studies indicated that the In_1_ site is the most favorable for substitution, having the lowest formation energy among all of the indium sites. Additionally, we identified the optimal configuration for embedding a Co_2_O_3_ cluster on the In_2_O_3_(111) surface. Both electron localization and Bader charge analyses revealed decreased ionic character around the active *b* oxygen site due to Co doping and Co_2_O_3_ embedding, which facilitates charge redistribution and enhances CO_2_ and H_2_ adsorption at the In2 and *b* sites. Additionally, Co modification of the In_2_O_3_ surface has been found to play a crucial role in stabilizing both reactants and products throughout the RWGS reaction, which offers a plausible explanation for recent experimental findings.^38^ To summarize, the present results on the energy and electronic properties of Co-modified In_2_O_3_ surfaces, along with the reported energy profiles for a plausible molecular mechanism of the RWGS reaction, provide valuable insights into advancing catalytic CO_2_ hydrogenation over doped In_2_O_3_ catalysts.

## Supplementary Material



## Notes

Figures S1–S8 reporting slab models for the low Miller index surfaces of In_2_O_3_; slab models with increased number of atomic layers and calculated Bader charges; a scheme illustrating the building and selection of models in this study, the different In_2_O_3_(111) surface models, and ELF analysis of In_2_O_3_(111) surface model; formation energy of Co-doped on the (111) surface of In_2_O_3_ and the possible configurations of Co_2_O_3_ clusters embedded into the In_2_O_3_ surface; the seven models of Co_2_O_3_ cluster embedded In_2_O_3_ (111) surface and the ELF analysis of In_2_O_3_(111) surface model (PDF)
