# Supplementary material for: Computational Investigation of Structure and Bonding of In2O3 Surfaces: Relevance to CO2 Hydrogenation
Source: J Phys Chem C Nanomater Interfaces. 2025 Jul 16;129(30):13713–22. doi: 10.1021/acs.jpcc.5c01539 (PMC12319902; doi:10.1021/acs.jpcc.5c01539)

Computational Investigation of Structure and Bonding of In_2O_3 Surfaces: Relevance to CO_2 Hydrogenation

Samadhan Kapse, Francesc Viñes, and Francesc Illas*

Departament de Ciència de Materials i Química Física & Institut de Química Teòrica i Computacional (IQTCUB), Universitat de Barcelona, c/ Martí i Franquès 1, 08028 Barcelona, Spain.

Content:

Figure S1: Slab models for the low Miller index surfaces of In_2O_3.

Figure S2: Slab models with increased number of atomic layers and calculated Bader charges.

Figure S3: Scheme illustrating the building and selection of models in this study.

Figure S4: The different In_2O_3(111) surface models.

Figure S5: ELF analysis of In_2O_3(111) surface model.

Figure S6: The formation energy of Co doped on the (111) surface of In_2O_3.

Figure S7: The possible configurations of Co_2O_3 clusters embedded into the In_2O_3 surface.

Figure S8: Represents the seven models of Co_2O_3 cluster embedded In_2O_3 (111) surface**.**

Figure S9: ELF analysis of In_2O_3(111) surface model.

Figure S1: Represents four slab models of (111), (110), (100)-O, and (100)-In surface of In_2O_3. The pink and red colour sphere indicates Indium and Oxygen atoms respectively.

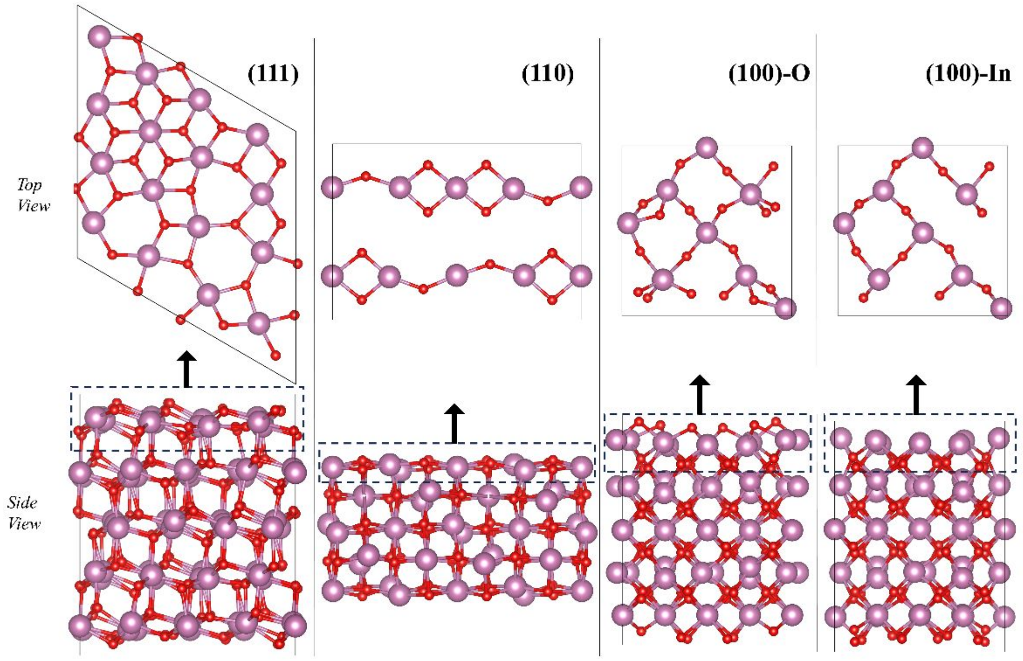

Figure S2: The In_2O_3 surface model of (111) with seven atomic layers, (110) with eight ionic layers, and (100)-O with eight ionic layers. The atoms with green and yellow-filled circles, represents the indium and oxygen atoms, respectively. The Bader charges for these highlighted atoms are provided in the table for each surface. Atomic colours as in Figure S1.

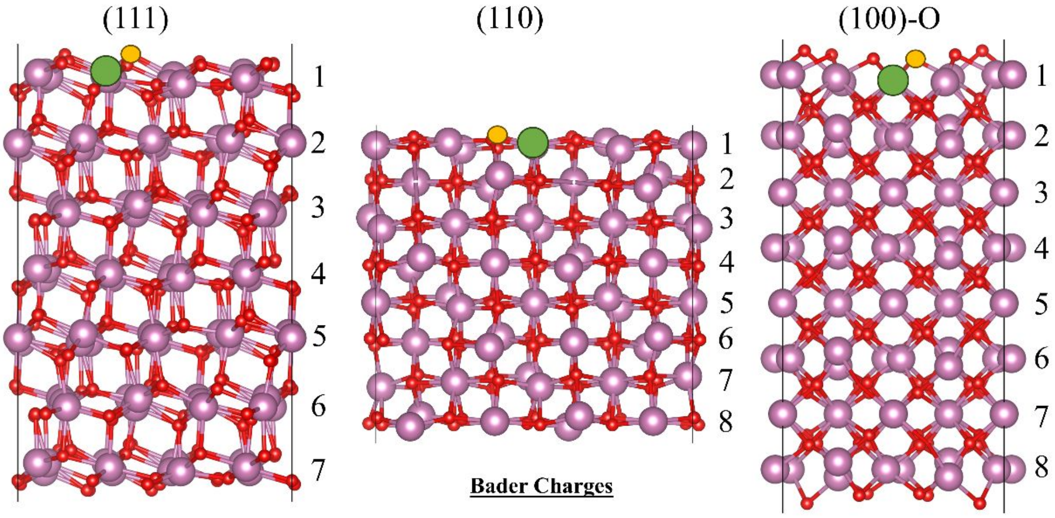

Bader Charges

(111)

Layers	Oxygen	Indium
3	-1.138	1.751
4	-1.140	1.751
5	-1.136	1.752
6	-1.136	1.752
7	-1.136	1.752

(110)

Layers	Oxygen	Indium
3	-1.122	1.744
4	-1.125	1.747
5	-1.128	1.745
6	-1.128	1.745
7	-1.129	1.745
8	-1.129	1.745

(100)-O

Layers	Oxygen	Indium
3	-1.101	1.708
4	-1.077	1.689
5	-1.069	1.675
6	-1.069	1.676
7	-1.069	1.674
8	-1.068	1.674

Figure S3: Scheme illustrating the building and selection of models used in this study. Colour coding as in Figure S1.

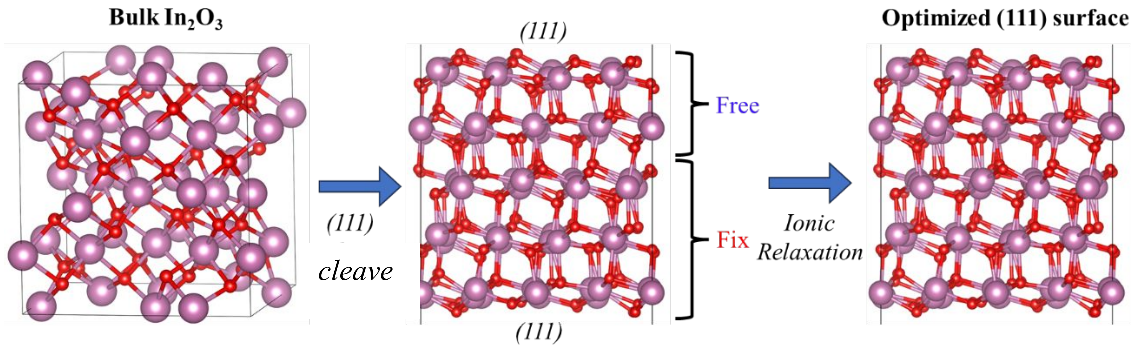

Figure S4: The In_2O_3 (111) surface models of fully relaxed, top and bottom relaxed, and top relaxed. Atomic colour-coding as in Figure S1.

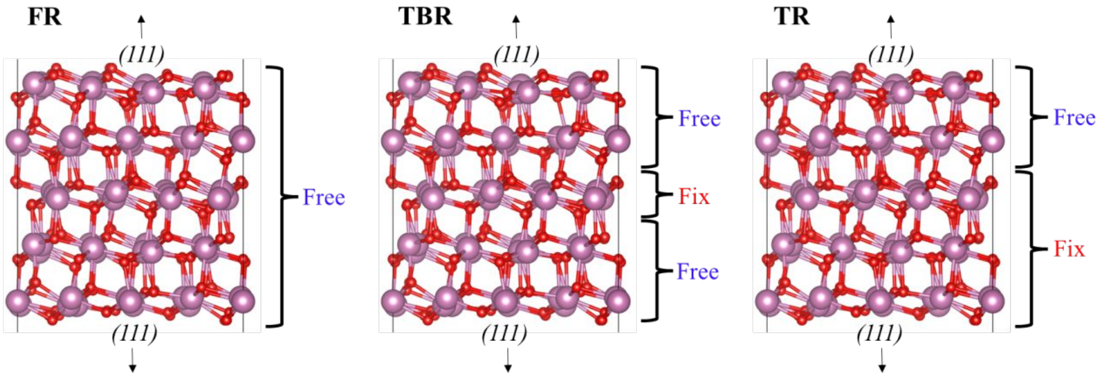

Figure S5: Electron localization function (ELF) of In_2O_3 (111) for the two different 2D slices shown in the figure. Atomic colour-coding as in Figure S1.

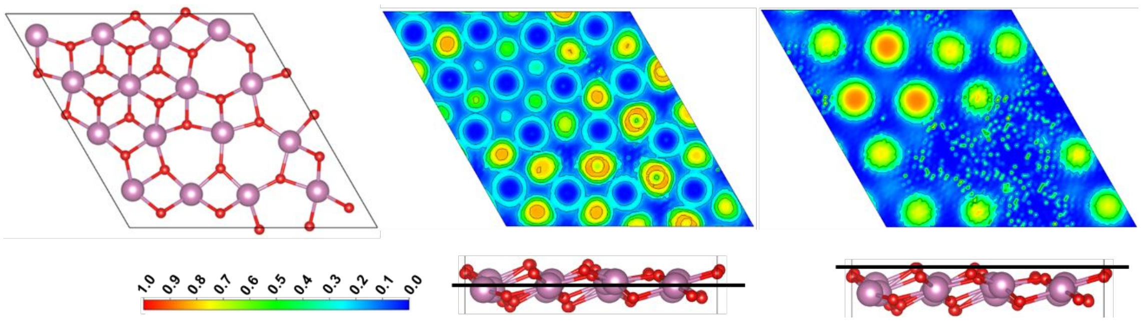

Figure S6: The formation energy, E_{form}, of Co doped at four indium positions on the (111) surface of In_2O_3.

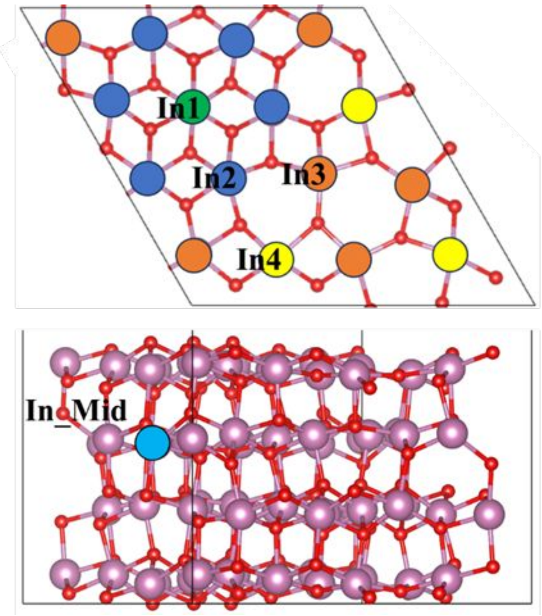

Co doped at	Energy	E_{form}
In1	-896.68	2.08
In2	-896.23	2.53
In3	-896.50	2.25
In4	-896.31	2.45
In_Mid	-896.05	2.69

Figure S7: Possible configurations of Co_2O_3 clusters embedded into the In_2O_3 surface. In each configuration, the three substituted surface In sites are marked by triangles of different colours. The structural model for configuration A is shown on the right.

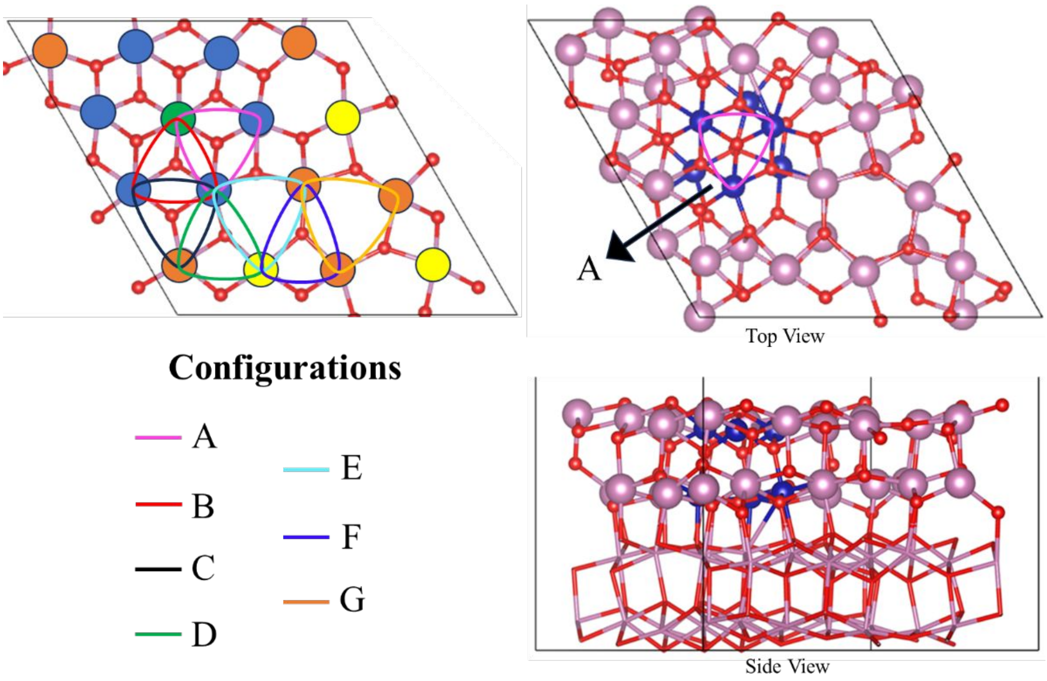

Figure S8: Represents the seven models of Co_2O_3 cluster embedded In_2O_3 (111) surface.

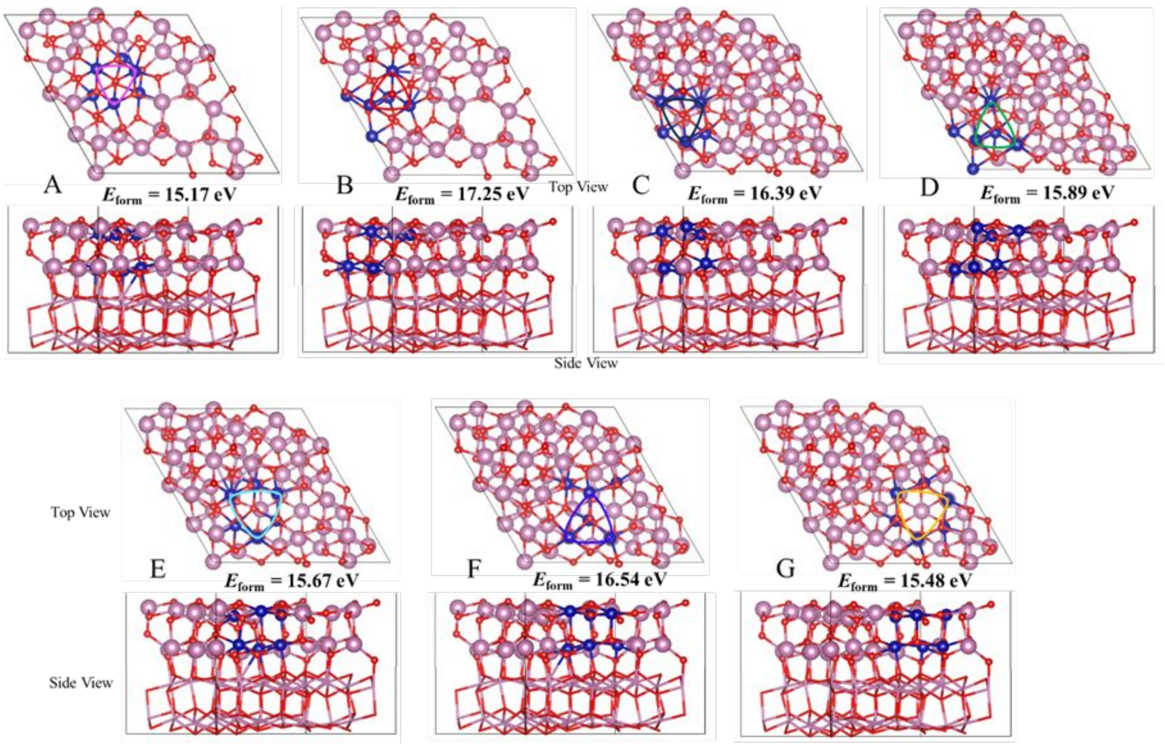

Figure S9: The ELF analysis for (111) surface model of stoichiometric, Co-doped, and Co_2O_3 cluster embedded In_2O_3.

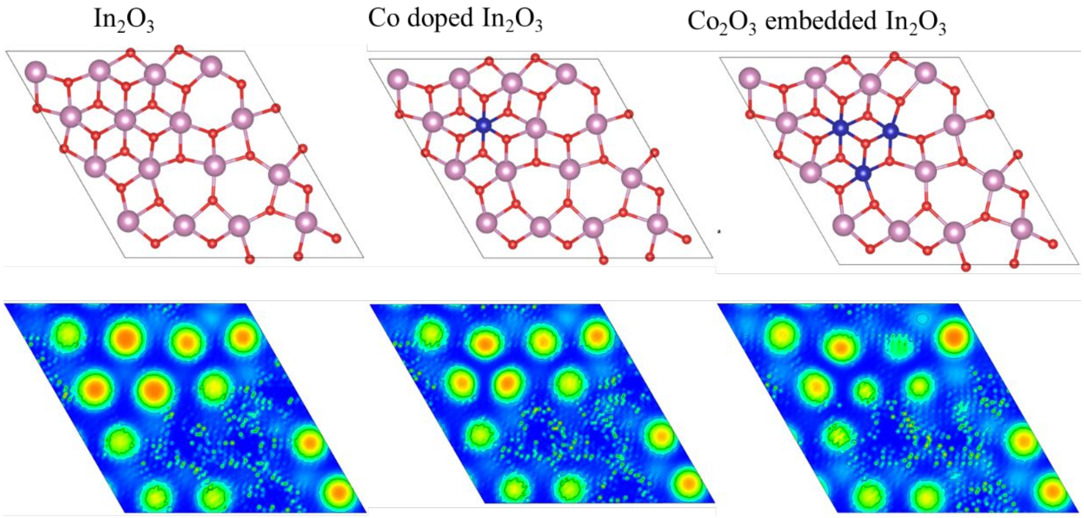

Supplement: Supplementary file 1 [file jp5c01539_si_001.pdf]
